# Rapid Detection of *Aspergillus fumigatus* Using Multiple Cross Displacement Amplification Combined With Nanoparticles-Based Lateral Flow

**DOI:** 10.3389/fcimb.2021.622402

**Published:** 2021-04-13

**Authors:** Luxi Jiang, Xiaomeng Li, Rumeng Gu, Deguang Mu

**Affiliations:** ^1^Department of Respiratory Medicine, Zhejiang Provincial People’s Hospital, People’s Hospital of Hangzhou Medical College, Hangzhou, China; ^2^Graduate School of Clinical Medicine, Bengbu Medical College, Bengbu, China

**Keywords:** *Aspergillus fumigatus*, multiple cross displacement amplification, nanoparticles-based biosensor, lateral flow biosensor, MCDA-LFB

## Abstract

*Aspergillus fumigatus* is an opportunistic, ubiquitous, saprophytic mold which can cause infection in the lungs, nose, eyes, brain, and bones in humans, especially in immunocompromised patients. However, it is difficult to diagnose *A. fumigatus* infection quickly. Here, we introduce a new detection method, namely multiple cross displacement amplification (MCDA) combined with nanoparticle-based lateral flow biosensor (LFB) (MCDA-LFB), which was proved to be fast, reliable, and simple for detecting *A. fumigatus*. We designed a set of 10 primers targeting the gene annexin ANXC4 of *A. fumigatus*. The best MCDA condition is 66 °C for 35 min. The minimum concentration that can be detected by this method was 10 fg. In the case of 100 sputum samples, 20 (20%) and 15 (15%) samples were positive by MCDA-LFB and PCR method, respectively. MCDA-LFB and traditional culture method showed the same results. Compared with the culture method, the diagnostic accuracy of MCDA-LFB can reach 100%. It showed that the MCDA-LFB method has better detection ability than the PCR method. We found that the whole process could be controlled within 60 min including the preparation of DNA (20 min), MCDA reaction (35 min) and results reporting (2 min). These results show that this assay is suitable for the rapid, sensitive and specific detection of *A. fumigatus* in clinical samples.

## Introduction

*Aspergillus fumigatus* is an opportunistic, ubiquitous, saprophytic mold, found in grain, contaminated food, soil and mold decay because it is one of the main pathogenic fungi in the world (Bultman et al., 2017; van de Veerdonk et al., 2017). It has a high morbidity and mortality rate, it can cause infection of the lungs, nose, eyes, brain, and bones in humans, especially in immunocompromised patients (Bakhshaee et al., 2016; Gamaletsou et al., 2017; Guest et al., 2018). *A. fumigatus* is the foremost reason of invasive aspergillosis such as invasive pulmonary aspergillosis (IPA), and invasive bronchial aspergillosis (IBA) and allergic disease (Wassano et al., 2020). It is also one of the most universal airborne fungal pathogens. In healthy individuals, the fungus is rapidly cleared by innate mechanisms, including immune cells (Moloney et al., 2016). However, in individuals with immunosuppression, *Aspergillus* spores can germinate into filamentous hyphae and invade and destroy tissues and organs and its mortality rates is as high as 50 to 60% in particular with invasive disease (Rosowski et al., 2018). Therefore, early and accurate diagnosis and treatment are crucial to the prognosis of patients.

Traditional methods used to identify *A. fumigatus* include direct microscopic examination, histopathology, culture, antigen test, and polymerase chain reaction (PCR). Each of them has its disadvantage. Direct microscopic examination has the advantages of rapid and effective, but it has poor sensitivity and is time-consuming. Besides, negative results cannot exclude other fungal infection (Zhang and Rickard, 2017). Histopathology is the golden standard for the diagnosis of *A. fumigatus*. However, this method is invasive, in some cases, biopsy is not possible due to the risk of bleeding, particularly in patients with hematologic abnormalities (McCarthy and Walsh, 2016). Culture needs special medium, and this method generally takes 3–10 days to develop and the positive rate is about 56% (Vergidis et al., 2020). PCR is an important method for the diagnosis of human pathogens including virus and bacterial infections. The diagnostic sensitivity is 88.5% (Zhang and Rickard, 2017). However, PCR-based-detection devices such as the Thermo Cycler, the Stratagene Mx3000P PCR (Stratagene, La Jolla, USA), or even the Light Cycler system (Roche, Mannheim, Germany) were so expensive that their use was limited in some less developed medical areas (Arastehfar et al., 2019)

In recent years, multiple cross displacement amplification (MCDA), a novel nucleic acid amplification technique, has been successfully used to detect a variety of pathogenic bacteria including *V. parahaemolyticus*, *Listeria monocytogenes* and *Salmonella* because of its high specificity, rapidity and simplicity (Wang et al., 2016a; Wang et al., 2016b; Wang et al., 2017). The reaction was implemented under isothermal conditions (61–68°C). Therefore, any instrument that can provide a constant temperature was feasible. In the analysis of MCDA products, lateral flow biosensors (LFBs) have been devised to visually detect double-labeled MCDA amplification within 1 to 2 min.

In this study, the MCDA-LFB assay for the detection of *A. fumigatus* was established based on the target sequence of *annexin ANXC4* gene (Khalaj et al., 2004). The sensitivity and specificity of the MCDA-LFB assay was evaluated, and 100 clinical sputum specimens were tested using this new method. Besides, we compared these results with the data from the culture and traditional PCR methods.

## Materials and Methods

### Agentias and Instrument

We got genomic template extraction kits (QIAamp DNA minikits; Qiagen, Hilden, Germany) from Qiagen (Beijing, China). Universal isothermal amplification kits and visual detection reagent (VDR) were obtained from Bei-Jing HaiTaiZhengYuan Co., Ltd. (Beijing, China). LFB materials, such as conjugate pad, sample pad, backing card, nitrocellulose membrane (NC) and absorbent pad were obtained from the Jie-Yi Biotechnology Co., Ltd. (Shanghai, China). Biotin-BSA (biotinylated bovine serum albumin) and anti-FITC (rabbit anti-fluorescein antibody), anti-dig (sheep anti-digoxigenin antibody) were purchased from Abcam Co., Ltd. (Shanghai, China). Dye (Crimson red) streptavidin-coated polymer nanoparticles (129 nm, 10 mg ml^−1^, 100 mM borate, pH 8.5 with 0.1% BSA, 0.05% Tween 20 and 10 mM EDTA) were obtained from Bangs Laboratories, Inc. (Indiana, USA).

### Bacterial and Fungi Strains, Culture Conditions and Genomic Template Preparation

We collected 66 strains (24 strains of *non-fumigating aspergillus* and 21 strains of bacteria from clinical and environmental samples in the study (**Table 1**). *A. fumigatus* (ATCC 96918) was used as reference strain to optimize the MCDA-LFB assay. DNA templates were extracted by the DNA extraction kit (QIAamp DNA Mini Kits, Hilden, Germany) under the guidance of the product instruction, and measured by a spectrophotometer (Nano drop ND-1000, Calibre, Beijing, China). The concentration of the target strain was continuously diluted by 10-fold (10 pg, 1 pg, 100 fg, 10 fg, 1 fg,100 ag, 10 ag) to optimize the temperature and test the sensitivity of the MCDA-LFB assay. And 1 μl of each dilution was used as template for MCDA reaction. The DNA extraction concentration of sputum samples used in this assay ranged from 7 to 58 ng/μl.

**Table 1:** Fungi and bacterial strains used in the current study.

Fungi	Strain no. (source of strains)^a^	No. of strains
*Aspergillus fumigatus*	^b^ATCC, ATCC96918	1
	Isolated strains (^c^ZJ)	20
*Cryptococcus neoformans*	ATCC13690	1
	Isolated strains (ZJ)	3
*Cryptococcus gattii*	Isolated strains (ZJ)	1
*Tropical candida*	Isolated strains (ZJ)	2
*Candida albicans*	ATCC10230	1
	Isolated strains (ZJ)	8
*Smooth candida*	Isolated strains (ZJ)	1
*Aspergillus Niger*	Isolated strains (ZJ)	1
*Aspergillus flavus*	Isolated strains (ZJ)	5
*Fusarium* spp.	Isolated strains (ZJ)	1
*Pseudomonas aeruginosa*	Isolated strains (ZJ)	1
*Acinetobacter bowman*	Isolated strains (ZJ)	8
*Penicillium Italium*	Isolated strains (ZJ)	1
*Dung enterococcus*	Isolated strains (ZJ)	1
*Proteus singularis*	Isolated strains (ZJ)	1
*S. aureus (^a^MRSA)*	ATCC 43300	1
	Isolated strains (ZJ)	6
*Streptococcus pharyngitis*	Isolated strains (ZJ)	1
*Enterobacter aerogenes*	Isolated strains (ZJ)	1

^a^MRSA, methicillin-resistant S. aureus. ^b^ATCC, American Type Culture Collection. ^c^ZJ, Zhejiang provincial people’s Hospital.

### Primer Reagent for MCDA Assay

**Figure 1:**
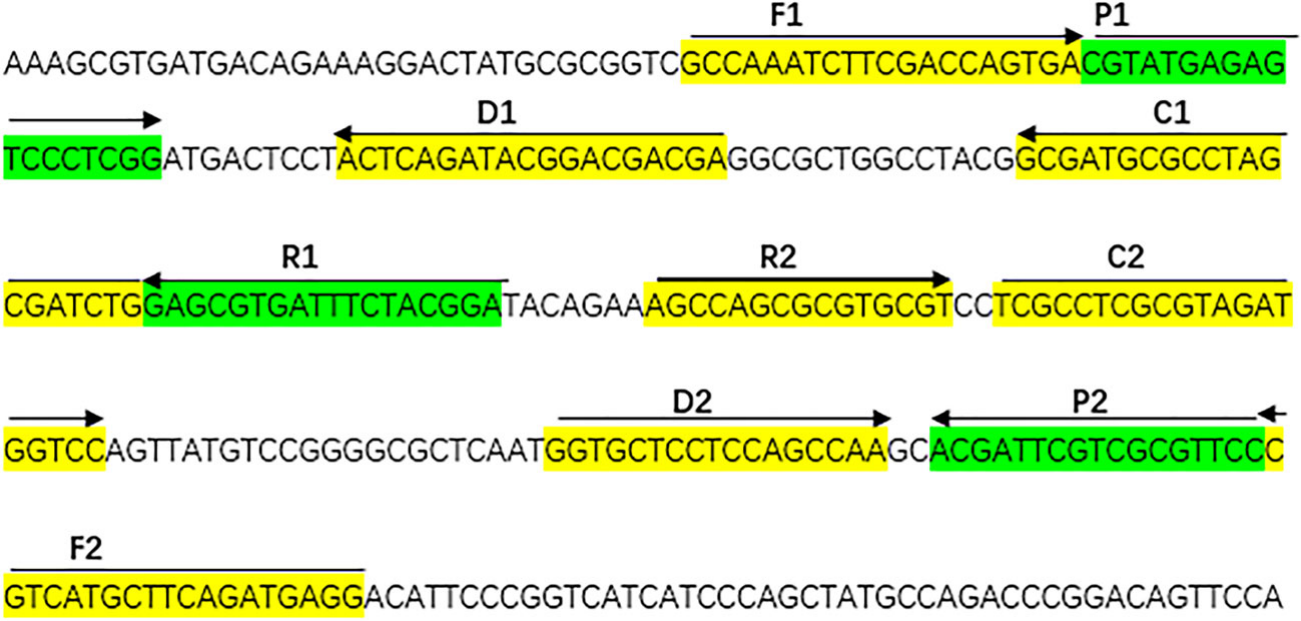
The sequences, positions and modifications of the *Aspergillus fumigatus*-specific gene *annex ANXC4*, Right arrows and left arrows indicate sense and complementary sequences that were used in the study.

With the help of Primer Explorer V4 (Eiken Chemical, Japan) and primer software PRIMER PREMIER 5.0, we designed specifically a set of 10 primers to recognize 10 different regions of the *A. fumigatus*-specific gene *annexin ANXC4*. The CP1 (C1 + P1) and C1 primers were labeled at their 5 end with biotin and fluorescein isothiocyanate (FITC), respectively. **Figure 1** and **Table 2** showed the sequences, positions and modifications of the MCDA primers used in the study. All primers were synthesized by TsingKe (Beijing, China) at HPLC purification grade.

**Table 2:** The primers used in the current report.

Primers name^a^	Sequences and modifications^b^	Length^c^	Genes
F1	5’-GCCAAATCTTCGACCAGTGA-3’	20 nt	
CP1	5’-CAGATCGCTAGGCGCATCGC-CGTATGAGAGTCCCTCGG-3’	39 mer	
CP1^∗^	5’-Biotin CAGATCGCTAGGCGCATCGC-CGTATGAGAGTCCCTCGG-3’	39 mer	
C1	5’-CAGATCGCTAGGCGCATCGC -3’	20 nt	*ANXC4*
C1^∗^	5’-FITC CAGATCGCTAGGCGCATCGC -3’	20nt	
D1	5’-TCGTCGTCCGTATCTGAGT-3’	19 nt	
R1	5’-TCCGTAGAAATCACGCTC-3’	18 nt	
R2	5’-AGCCAGCGCGTGCGT-3’	15 nt	
D2	5’-GGTGCTCCTCCAGCCAA-3’	17 nt	
C2	5’-TCGCCTCGCGTAGATGGTCC-3’	20 nt	
CP2	5’-TCGCCTCGCGTAGATGGTCC-GGAACGCGACGAATCGT-3’	38 mer	
F2	5’-CCTCATCTGAAGCATGACG-3’	19 mer	

^a^CP1∗ 5’-labeled with biotin when used in MCDA-LFB assay.

^a^C1∗ 5’-labeled with FITC when used in MCDA-LFB assay.

^b^FITC, fluorescein isothiocyanate.

^c^mer, monomeric unit; nt, nucleitide.

### Preparation and Operation of Lateral Flow Biosensor (LFB)

Lateral flow biosensor (LFB) was used to detect the results of the MCDA assay according to previous literature reports (Wang et al., 2016a; Wang et al., 2016b; Wang et al., 2017). Generally speaking, the biosensor is made up of a backing pad, an immersion pad, a conjugate pad, NC membrane, and an absorbent pad. The capture reagents were fixed in 0.01 M phosphate-buffered saline (PBS, Ph 7.4), and the anti-FITC Ab (0.2 mg/ml) and biotin-BSA(4 mg/ml) conjugates were sprayed onto NC membrane to form two lines. The first line is the test line (TL), the second line is the control line (CL), with each line separated by 5 mm. SA-PNP (Dye streptavi-din-coated polymer nanoparticles, 129 nm, 10 mg ml^−1^,100 mM borate, pH 8.5 with 0.1% BSA, 0.05% Tween 20 and 10mM EDTA) was deposited on the conjugate pad.

### The MCDA Assay

Based on previous studies (Wang et al., 2016a; Wang et al., 2016b; Wang et al., 2017), the 25 µl mixtures of the MCDA assays were consist of 0.1 µl each F1 and F2 primers, 0.2 µl each C1∗ and C2 primers, 0.2 µl each R1,R2, D1 and D2 primers, 0.4 µl each CP1 and CP1∗primers, 0.4 µl each CP2 primer, 1 µl DNA template, 12.5 µl (2 ×) buffer (Universal isothermal amplification kits) and 1 µl (8 U) of *Bst* DNA polymerase. All the mixtures were heated for 35 min at 66°C. *Cryptococcus neoformans* (ATCC13690) and *Candida albicans* strains (ATCC10230) were used as negative controls (NC), and double distilled water (DW) were used as a blank control.

### Optimizing the Reaction Temperature of MCDA-LFB Assay

It was important to detect the optimal temperature of the MCDA-LFB assay. The 25 µl mixtures of the MCDA assay was placed at different reaction temperature (61–68°C, with 1°C interval) to optimize the amplification temperature. We used real-time turbidity detection and the kinetic curves at different temperatures to analyze the reactions of the MCDA assay.

### Sensitivity of MCDA-LFB Assay

The concentration of the target DNA was continuously diluted by 10-fold (10 pg, 1 pg, 100 fg, 10 fg, 1 fg, 100 ag, 10 ag) to test the sensitivity of the MCDA-LFB assay. We added 1 μl of the above diluted concentration and DW into the amplification mixtures. The LoD (limit of detection) was considered to be the dilution gradients that finally show a positive result.

### Optimizing the Reaction Time of MCDA-LFB Assay

The optimal time of the MCDA-LFB assay was valuated at different times (from 25 to 55 min, with 10 min intervals). We reported the results by LFB.

### Specificity of MCDA-LFB Assay

We proved the specificity of this MCDA-LFB assay by genomic DNA (at least 10 ng per microliters) from 66 strains (**Table 1**). And we also used lateral flow biosensor (LFB) to show the results of all MCDA assays and observe the color change. The assay was repeated for three times.

### Preparation of Sputum Specimens

We cooperated with the Department of Laboratory Medicine. After collection, DNA templates from sputum samples were directly extracted using the protocol of QIAamp and then labeled and stored in the refrigerator at −20°C in the study.

### Evaluation of the Feasibility of MCDA-LFB Assay

A total of 100 sputum samples, which were suspected from human *A. fumigatus*, were collected from Zhejiang province, China (Ethic approval: The research protocol for the current study has been approved by The Ethics Committee of the Zhejiang provincial people’s Hospital, Hangzhou, Zhejiang, China). These samples were used for *A. fumigatus* diagnosis using culture-based method, traditional PCR method, and this new MCDA-LFB assay. Traditional cultures were conducted with Sabouraud Dextrose Agar (SDA). In short, about 1 ml sputum samples were inoculated into a fungal chromogenic plate to culture under suitable conditions for 3–10 days, and then the fungi strain was selected for culture. DNA templates from samples (500μL) were directly extracted using protocol of QIAamp for PCR and MCDA-LFB assays. PCR was performed with *A. fumigatus* specific primers for *annexin ANXC4* gene with an amplicon size of 415 bp (Khalaj et al., 2004). And finally, we compared the results of MCDA-LFB method with those of the culture and PCR methods.

## Results

### Confirmation and Detection of *Aspergillus fumigatus* MCDA Products

**Figure 2:**
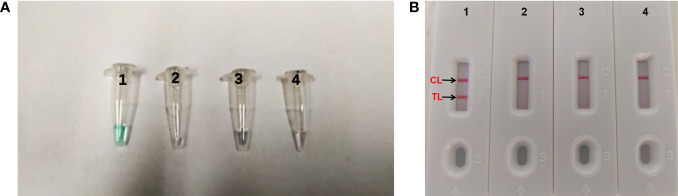
Confirmation and detection of *Aspergillus fumigatus*-MCDA products. **(A)** Color change of *A fumigatus*-MCDA tubes; **(B)** (right), LFB applied for visual detection of *A. fumigatus*-MCDA products. Tube A1, positive amplification; tube A2, negative amplification (*Cryptococcus neoformans*), tube A3 negative amplification (*Candida albicans*), tube A4, negative control (DW); Biosensor B1, positive amplification; biosensor B2, negative amplification (*Cryptococcus neoformans*), biosensor B3, negative amplification (*Candida albicans*), biosensor B4, negative control (DW).

When the assay was conducted at a fixed temperature of 66°C for 1 h. The DNA of *A. fumigatus* (ATCC96918) was amplified, but the DNA of *Cryptococcus neoformans* (ATCC13690), *Candida albicans* strains (ATCC10230) and the DW control was not (**Figure 2**). The *A. fumigatus*-MCDA amplified products presented light green, while colorlessness was remained in the negative ones (**Figure 2A**). For LFB, two red bands, including TL and CL, appeared on the biosensors for the positive amplification, and a single red band (CL) appeared on the biosensors for negative and blank controls (**Figure 2B**). Therefore, the primer based on the *annexin ANXC4* gene was very suitable for the establishment of the MCDA-LFB assay.

### Optimal Temperature of MCDA Primer Set

**Figure 3:**
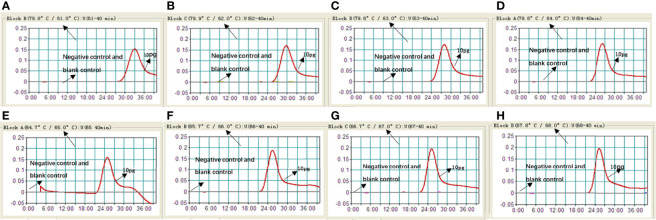
Optimizing the reaction temperature of MCDA-LFB assay. We used real-time turbidity detection, and the kinetic curves at different temperatures to analyse the reactions of the MCDA assay. The threshold value was 0.1 and the turbidity of >0.1 was considered to be positive. Eight kinetic graphs **(A–H)** were generated at various temperatures (61–68°C, 1°C intervals) with target pathogens DNA at the level of 10 pg per reaction.

It is important to detect the optimal temperature of the MCDA-LFB assay. We used target DNA (10 pg/ml) at different reaction temperature (61–68°C, with 1°C interval) to optimize the amplification temperature. We used real-time turbidity detection, and the kinetic curves at different temperatures to examine the reactions of the MCDA assay (**Figure 3**). In the end, the optimal temperature for the MCDA assay was 66°C.

### Specificity of *Aspergillus fumigatus*-MCDA-LFB Assay

**Figure 4:**
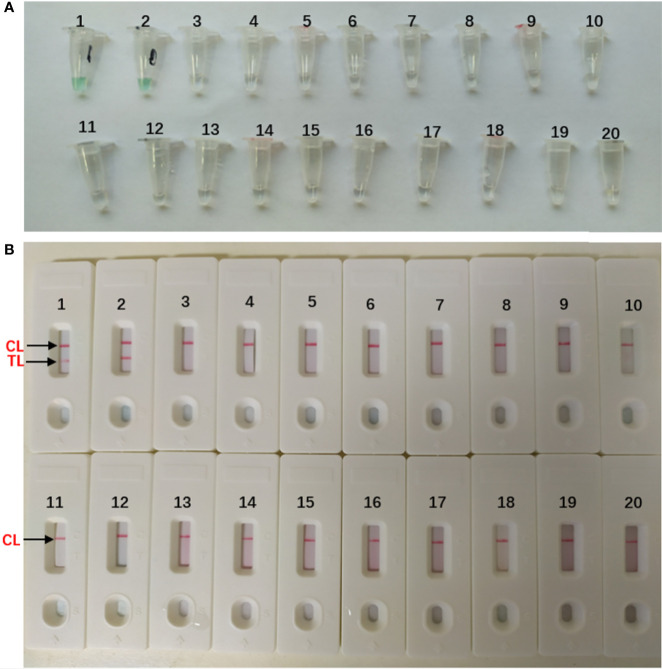
Specificity of *Aspergillus fumigatus*-MCDA-LFB assay **(A)** Color change of *A fumigatus*-MCDA tubes; **(B)** LFB applied for visual detection of *A. fumigatus*-MCDA products. The MCDA assay were conducted using different genomic DNA templates and these results were analyzed visually. TubeA1, *Aspergillus fumigatus* (ATCC96918); Tube A2, isolated strains of *Aspergillus fumigatus*; Tube A3-18, *Cryptococcus neoformans* (ATCC13690); *Cryptococcus gattii*; *Tropical candida*; *Candida albic* (ATCC10230); *Smoothcandida*; *Aspergillus niger; Aspergillusflavus*; *Fusarium* spp; *Pseudomonasaeruginosa*; *Acinetobacterbowman*; *Penicillium italium*; *Dungenterococcus*; *Proteus singularis*; *S. aureus(*ATCC 43300); *Streptococcus pharyngitis*; *Enterobacter aerogenes*. Tube A19-20, blank control (DW). BiosensorB1, *Aspergillus fumigatus* (ATCC96918); BiosensorB2, isolated strains of *Aspergillus fumigatus*; Biosensor B3-18, negative amplification (The strain is the same as Tube A3-18); BiosensorB19-20, negative control (DW).

The DNA templates of the collected strains (**Table 1**) were used to determine the specificity of the MCDA-LFB assay. All the *A. fumigatus* strains showed positive results. We could see two red lines (TL and CL) on the biosensors and presented light green [**Figures 4(B1–2, A1–2)**]. And non-*A. fumigatus strains*, blank control appeared negative results and presented colorlessness [**Figures 4(A3–20)**]. Only a red line (CL) showed on the biosensors [**Figures 4(B3–20)**].

### Sensitivity of *Aspergillus fumigatus*-MCDA-LFB Assay

**Figure 5:**
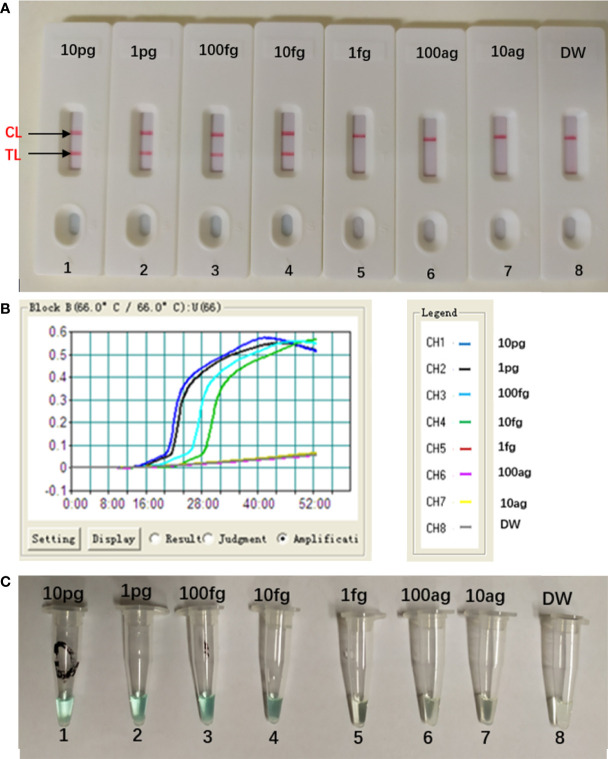
Sensitivity of *Aspergillus fumigatus*-MCDA-LFB assay. Serially diluted genomic templates of *Aspergillus fumigatus* strain (ATCC96918) were used to detect the sensitivity of the MCDA-LFB assay. The LoD (limit of detection) was analyzed by LFB **(A)** and real time turbidity **(B)**, colorimetric indicator detection **(C)**. The serial dilutions (10 pg, 1 pg, 100 fg, 10 fg, 1 fg and 100 ag, 10 ag) of target templates were used for standard MCDA reactions. Biosensors **(A)** Turbidity signals **(B)** Tubes **(C)** 1–8 represented the DNA levels of 10 pg (*Aspergillus fumigatus* ATCC96918 templates), 1 pg (*Aspergillus fumigatus* ATCC96918 templates), 100 fg (*Aspergillus fumigatus* ATCC96918 templates), 10 fg (*Aspergillus fumigatus* ATCC96918 templates), 1 fg (*Aspergillus fumigatus* ATCC96918 templates) and 100 ag (*Aspergillus fumigatus* ATCC96918 templates), 10 ag (*Aspergillus fumigatus* ATCC96918 templates) and blank control (DW). The genomic DNA levels of 10 pg to 10 fg per reaction produced the positive results.

The concentration of the target DNA was continuously diluted by 10-fold (10 pg, 1 pg, 100 fg, 10 fg, 1 fg, 100 ag, 10 ag) to test the sensitivity of the MCDA-LFB assay. As seen in **Figure 5**, the LoD of this assay was 10 fg per reaction, and two crimson lines (CL and TL) appeared on the LFB, indication that the target gene was positive (**Figure 5A**). This result was in consistent with the real-time turbidity detection (**Figure 5B**) and colorimetric indicator detection (**Figure 5C**).

### Optimal Duration Time for *Aspergillus fumigatus*-MCDA-LFB Assay

**Figure 6:**
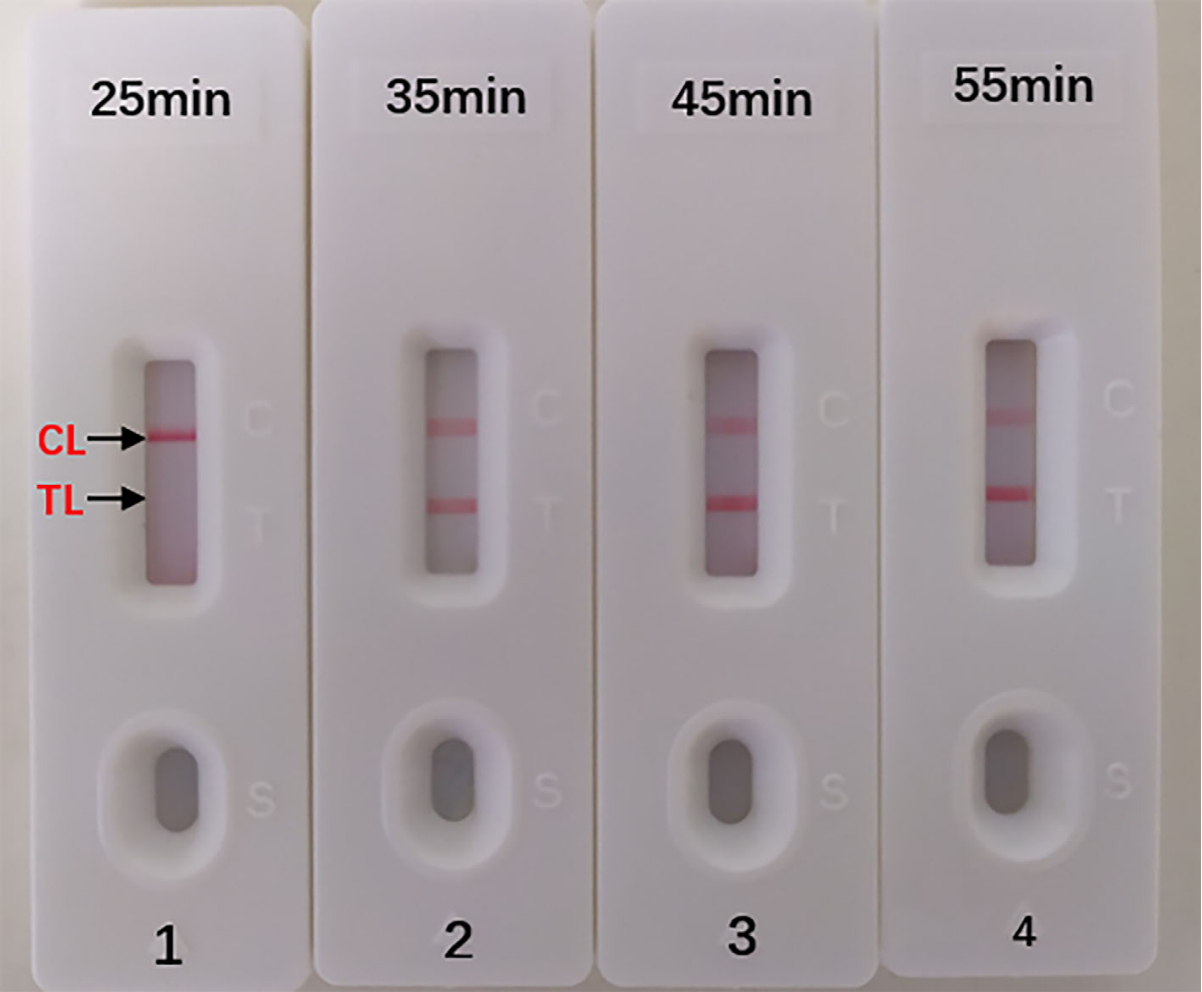
Optimal Duration Time for *Aspergillus fumigatus*-MCDA-LFB assay. Four different reaction times (1) 25 min; (2) 35 min; (3) 45 min; (4) 55 min) were tested and compared at 66 °C. Biosensors 1, 2, 3, 4 represent DAN levels of 10 fg/μl (LoD level). The best time was 35 min.

The optimal duration time of the MCDA-LFB assay was evaluated at different duration time (from 25 to 55 min, with 10 min intervals) with the optimal amplification temperature (66°C). And the target DNA at the LoD level (10 fg) could be detected when MCDA-LFB reaction lasted only for 35 min (**Figure 6**).

### Demonstrating the Feasibility of MCDA-LFB Assay by Sputum Samples

In order to further confirm the feasibility of MCDA-LFB assay. We tested 100 sputum specimens with high suspicion of fungal infection using culture, PCR and MCDA-LFB assay. DNA templates from 100 samples were directly extracted using the protocol of QIAamp and used for PCR and MCDA-LFB assays. The results were shown in **Table 3**. Among 100 sputum samples, 20 (20%) and 15 (15%) samples were positive by MCDA-LFB and PCR method, respectively. In conclusion, compared with the culture method, the diagnostic accuracy of MCDA-LFB can reach 100%. From these results we could conclude that the MCDA-LFB assay is an efficient examination to diagnose *A. fumigatusis* in clinical samples.

**Table 3:** Comparison of PCR, culture-biotechnical, and MCDA-LFB assays for the detection of *Aspergillus fumigatus* in sputum samples of human.

Detection methods	Whole sputum samples (n = 100)	
	Positive	Negative
MCDA-LFB	20 (20%)	80
Culture	20 (20%)	80
PCR	15 (15%)	85

## Discussion

*A. fumigatus* is a conditioned fungal pathogen, which can cause severe lesions when the body’s immunity is low (Watkins et al., 2018). *A. fumigatus* is the most common isolate of invasive aspergillus (IA) (Wassano et al., 2020). Its fatality rate is as high as 50% (Zhang et al., 2017). The low sensitivity of fungal culture can easily lead to missing diagnosis. So how to diagnose *A. fumigatus* infection quickly brings great challenge to clinical workers. It is urgent to find an efficient detection method. We finally established MCDA assay which can distinguish *A. fumigatus* with the help of the specific gene *annexin ANXC4*. Then, we used the specific gene *annexin ANXC4* to design a set of 10 primers (**Figure 1** and **Table 2**). The specificity of this assay was successfully tested using collected strains (20 strains of *A. fumigatus* and 45 strains of non-*A. fumigatus*). We could see two red lines (TL and CL) on the biosensors and presented light green for all *A. fumigatus*. but only one red line on the biosensors and presented colorlessness for all non-*A. fumigatus* (**Figure 4**). These results indicated that the MCDA-LFB was highly selective in identifying *A. fumigatus*.

In addition to specificity, the MCDA-LFB assay also has high sensitivity. The MCDA-LFB assay can distinguish a minimum concentration of 10 fg (**Figure 5**). And LFB is widely used because of its simplicity, convenience and quickness during the entire process of the experiment. When LFB compared with other detection methods (real time turbidity, colorimetric indicator detection), LFB can judge the results more intuitively, quickly and accurately (**Figures 2**, **4**, **5**). Besides, colorimetric indicator detection is prone to misjudgment because its subjectivity is too strong.

In order to further prove the practical value of the MCDA-LFB assay, 100 sputum specimens with high suspicion of fungal infection were verified by MCDA-LFB, culture and PCR respectively. From **Table 3**, we could see that the MCDA-LFB assay show a high sensitivity. Five of the sputum specimens were tested positive for MCDA-LFB and culture tests, but tested negative by traditional PCR test. The reason is probably because the copy number of target gene template of the samples can’t meet the limit of detection of the PCR assay. By contrast, the MCDA-LFB assay can not only save time but also improve the accuracy of detection, and the required instrument (an instrument which can provide a constant temperature of 66°C) is relatively simple.

All in all, the MCDA-LFB assay could be controlled within 60 min including the preparation of DNA (20 min), MCDA reaction (35 min) and results reporting (2 min). The MCDA-LFB assay greatly saved time and improved efficiency in doing clinical diagnosis compared with the culture method and the PCR method.

## Conclusion

In conclusion, we succeed in establishing the MCDA-LFB detection method which based on the *annexin ANXC4* genes. This method showed high specificity and high sensitivity, which could successfully detect *A. fumigatus* isolates, and had the LoD of 10fg genomic template per tube. Besides, the clinical effectiveness of this assay was successfully upheld by sputum samples. In brief, the MCDA-LFB detection method is suitable for the rapid, sensitive and specific detection of *A. fumigatus* and it can become a new method in basic and clinical laboratories.

## Data Availability Statement

The raw data supporting the conclusions of this article will be made available by the authors, without undue reservation.

## Ethics Statement

The studies involving human participants were reviewed and approved by The Ethics Committee of the Zhejiang provincial people’s Hospital. The patients/participants provided their written informed consent to participate in this study.

## Author Contributions

DM and LJ conceived and designed the experiments. XL and RG performed the experiments. DM, LJ, XL, and RG contributed the reagents and materials and analyzed the data. XL wrote the manuscript. DM and LJ reviewed the manuscript. All authors contributed to the article and approved the submitted version.

## Funding

This study was supported by grants from Medical Science and Technology Project of Zhejiang province (No. 2020KY400).

## Conflict of Interest

The authors declare that the research was conducted in the absence of any commercial or financial relationships that could be construed as a potential conflict of interest.
