# Supplementary material for: Rapid Detection of Aspergillus fumigatus Using Multiple Cross Displacement Amplification Combined With Nanoparticles-Based Lateral Flow
Source: Front Cell Infect Microbiol. 2021 Apr 13;11:622402. doi: 10.3389/fcimb.2021.622402 (PMC8076636; doi:10.3389/fcimb.2021.622402)
Supplement: Supplementary file 1 [file Table_1.docx]

**Table S1**

| Collected strain | Culture | PCR | MCDA-LFB |
| --- | --- | --- | --- |
| strain 1 | + | + | + |
| strain 2 | + | + | + |
| strain 3 | + | + | + |
| strain 4 | + | + | + |
| strain 5 | + | + | + |
| strain 6 | + | + | + |
| strain 7 | + | + | + |
| strain 8 | + | + | + |
| strain 9 | + | + | + |
| strain 10 | + | + | + |
| strain 11 | + | + | + |
| strain 12 | + | + | + |
| strain 13 | + | + | + |
| strain 14 | + | + | + |
| strain 15 | + | + | + |
| strain 16 | + | + | + |
| strain 17 | + | + | + |
| strain 18 | + | + | + |
| strain 19 | + | + | + |
| strain 20 | + | + | + |
| strain 21 | + | + | + |
| strain 22 | + | + | + |
| strain 23 | + | + | + |
| strain 24 | + | + | + |
| strain 25 | + | + | + |
| strain 26 | + | + | + |
| strain 27 | + | + | + |
| strain 28 | + | + | + |
| strain 29 | + | + | + |
| strain 30 | + | + | + |
| strain 31 | + | + | + |
| strain 32 | + | + | + |
| strain 33 | + | + | + |
| strain 34 | + | + | + |
| strain 35 | + | + | + |
| strain 36 | + | + | + |
| strain 37 | + | + | + |
| strain 38 | + | + | + |
| strain 39 | + | + | + |
| strain 40 | + | + | + |
| strain 41 | + | + | + |
| strain 42 | + | + | + |
| strain 43 | + | + | + |
| strain 44 | + | + | + |
| strain 45 | + | + | + |
| strain 46 | + | + | + |
| strain 47 | + | + | + |
| strain 48 | + | + | + |
| strain 49 | + | + | + |
| strain 50 | + | + | + |
| strain 51 | + | + | + |
| strain 52 | + | + | + |
| strain 53 | + | + | + |
| strain 54 | + | + | + |
| strain 55 | + | + | + |
| strain 56 | + | + | + |
| strain 57 | + | + | + |
| strain 58 | + | + | + |
| strain 59 | + | + | + |
| strain 60 | + | + | + |
| strain 61 | + | + | + |
| strain 62 | + | + | + |
| strain 63 | + | + | + |
| strain 64 | + | + | + |
| strain 65 | + | + | + |
| strain 66 | + | + | + |
| strain 67 | + | + | + |
| strain 68 | + | + | + |
| strain 69 | + | + | + |
| strain 70 | + | + | + |
| strain 71 | + | + | + |
| strain 72 | + | + | + |
| strain 73 | + | + | + |
| strain 74 | + | + | + |
| strain 75 | + | + | + |
| strain 76 | + | + | + |
| strain 77 | + | + | + |
| strain 78 | + | + | + |
| strain 79 | + | + | + |
| strain 80 | + | + | + |
| strain 81 | + | + | + |
| strain 82 | + | + | + |
| strain 83 | + | + | + |
| strain 84 | + | + | + |
| strain 85 | + | + | + |
| strain 86 | + | + | + |
| strain 87 | + | + | + |
| strain 88 | + | + | + |
| strain 89 | + | + | + |
| strain 90 | + | + | + |
| strain 91 | + | + | + |
| strain 92 | + | + | + |
| strain 93 | + | + | + |
| strain 94 | + | + | + |
| strain 95 | + | + | + |
| strain 96 | + | + | + |
| strain 97 | + | + | + |
| strain 98 | + | + | + |
| strain 99 | + | + | + |
| strain 100 | + | + | + |
